# Assessment of Choroidal Microstructure and Subfoveal Thickness Change in Eyes With Different Stages of Age-Related Macular Degeneration

**DOI:** 10.1097/MD.0000000000002967

**Published:** 2016-03-11

**Authors:** Linna Lu, Shiqiong Xu, Fangling He, Yan Liu, Yidan Zhang, Jing Wang, Zhiliang Wang, Xianqun Fan

**Affiliations:** From the Department of Ophthalmology, Ninth People's Hospital, Shanghai Jiaotong University School of Medicine, Shanghai, China.

## Abstract

Age-related macular degeneration (AMD) is a major cause of irreversible blindness. Choroidal structural changes seem to be inevitable in AMD pathogenesis. Our study revealed associated choroidal microstructural changes in AMD eyes.

The aim of the study was to compare choroidal microstructural changes in eyes with AMD of different stages.

The study was a retrospective, cross-sectional case series.

The participants comprised of 32 age-matched normal eyes as controls, and 26 fellow uninvolved eyes of intermediate/late AMD, 29 of early AMD, 28 of intermediate AMD, and 39 of late AMD.

All subjects underwent comprehensive ophthalmologic examination. The choroid images, including subfoveal choroidal thickness, percentage of Sattler layer area, and en face images of the choroid, were obtained using spectral-domain optical coherence tomography.

The main outcome measures were subfoveal choroidal thickness changes, percentage of Sattler layer area changes, and en face images of the choroid in AMD eyes.

One hundred fifty-four eyes of 96 individuals with mean age of 67.1±9.2 years were included. The mean subfoveal choroidal thickness was 295.4 ± 56.8 μm in age-matched normal eyes, 306.7 ± 68.4 μm in fellow uninvolved eyes with AMD, 293.8 ± 80.4 μm in early AMD, 215.6 ± 80.4 μm in intermediate AMD, and 200.4 ± 66.6 μm in late AMD (*F* = 14.2, all *P* < 0.001). Choroidal thickness was greater in early AMD eyes than in intermediate/late AMD eyes (*P* < 0.001). Mean percentage of Sattler layer area in each group showed a similar tendency. Microstructure of the choroid showed reduced vascular density of Sattler layer areas in late AMD eyes compared with normal eyes.

Decreasing subfoveal choroidal thickness and percentage of Sattler layer area were demonstrated in the progression of AMD. The choroidal change was related to atrophy of the microstructural changes of underlying capillaries and medium-sized vessels.

## INTRODUCTION

Age-related macular degeneration (AMD) is a major cause of irreversible blindness in developed and developing countries, particularly in people older than 60 years.^1–3^ There are currently several AMD classification schemes that include the terms nonexudative and exudative AMD,^4,5^ which correlate with visual acuity. Several histological changes can be useful to make the clinical diagnosis, but some of them have different progression characteristic associated with their dissimilar causes and risk factors (genetic and phenotypic). In 2013, the Beckman Initiative for Macular Research Classification Committee provided a consensus recommendation clinical classification system of the AMD phenotype for either clinical or research purposes. The committee was in agreement that the terms wet and dry AMD were problematic because dry can include simple drusen to geographical atrophy (GA) or even old disciform scars formation. Therefore, a new AMD classification was suggested: those with no visible drusen or pigmentary abnormalities are considered to have no signs of AMD; those with drusen <63 μm are considered to have normal aging changes; drusens between 63 and 125 μm without pigmentary abnormalities are early AMDs; drusen >125 μm or with pigmentary abnormalities associated with at least medium drusen is intermediate AMD, whereas those with neovascular AMD or GA are late AMD. In this new classification, they limited the term dry to GA and did not refer to earlier stages of AMD as dry. The proposed basic clinical classification scale seems to be of value in predicting the AMD risk.^6^ The choroid requires high blood flow to satisfy the normal metabolic demands of oxygen supply to the outer retina. In nonexudative and exudative AMD patients, an intimate relationship has been identified between retinal pigment epithelial atrophy and choroidal circulation defects, especially of the choriocapillaris.^7,8^ In AMD, histological evidence of a loss of the choriocapillaris has also been demonstrated.^9^ Thus, choroidal structural changes seem to be inevitable in AMD pathogenesis. Significant advances of enhanced depth imaging spectral-domain optical coherence tomography (SD-OCT) and recent use of “en face” OCT allow a cross-sectional, noninvasive, layer-by-layer visualized reconstructed image of the involved retina and choroid in vivo. Using SD-OCT, studies have shown choroidal changes in different diseases, such as central serous chorioretinopathy (CSCR),^10–12^ high myopia,^13^ and pseudoxanthomaelasticum (PXE).^14^ Changes in the choroid, in different types of AMD patients, have also been documented,^15–17^ suggesting that reduced choroidal thickness may play an indirect role as an indicator of the natural history of AMD. However, the associations between the choriocapillaris changes and pathogenesis of the progression of AMD remain controversial.^18,19^

In this study, we investigated a consecutive series of patients with different stages of AMD based on the 2013 consensus of The Beckman Initiative for Macular Research Classification Committee, to elucidate the choroidal microstructural changes.

## METHODS

This was a single-center, retrospective, cross-sectional case series, performed at the Department of Ophthalmology, Ninth People's Hospital, Shanghai JiaoTong University School of Medicine, between January 14, 2014 and July 15, 2014. Approval was obtained from the Institute's ethics committee, the study was conducted in accordance with the tenets of the Declaration of Helsinki, and patient consent was obtained.^20^

### Subjects

All individuals included were >55 years old and underwent a comprehensive ophthalmologic examination and routine diagnostic evaluation consisting of best-corrected visual acuity, color fundus photography, fundus fluorescence angiography (FFA), and optical coherence tomography (OCT). Fellow uninvolved eyes of intermediate and late AMD patients have been found to have predictive risk factors for progressive late AMD. Subjects with additional retinal pathologies known to affect choroidal thickness, other than AMD, or with previous intraocular surgeries including cataract surgery, vitrectomy, or having undergone antivascular endothelial growth factor (VEGF) therapy within the previous 3 months, or eyes with histories of ocular diseases, such as glaucoma, ocular inflammation, retinal detachment, refractive errors >3.0 diopters, ocular diseases associated with systemic diseases such as diabetic retinopathy, arteriosclerotic retinopathy, and diseases that obstruct the images of the choroid, such as cataract or subfoveal hemorrhage, were excluded. GA patients were excluded because of its different mechanism and treatment from wet AMD. Polypoidal choroidal vasculopathy (PCV) eyes were also excluded because of the different pathophysiological features when compared with general wet AMD.

According to the consensus made in 2013,^6^ individuals with no visible drusen and small drusen (<63 μm) should be considered without clinically relevant increased risk of late AMD development, so we put these individuals under the category of age-matched normal eyes serving as controls. Furthermore, the fellow eye of individuals who already have late AMD should be estimated separately because the other eye was affected significantly by the status of the fellow eye. Early and intermediate AMDs which have different relevance to risk of disease progression also need to be discussed. We divided the patients into 5 groups after examinations of color fundus photography, OCT, and FFA images. Group 1 (G1) included eyes with no clinically relevant increased risk of late AMD developing; group 2 (G2) included fellow uninvolved eyes of AMD patients; group 3 (G3), as the early AMD eyes, was characterized by early AMD (medium drusen >63 μm and ≤125 μm; group 4 (G4), as intermediate AMD, was characterized with large drusen ≥125 μm; and group 5 (G5) included late AMD eyes, characterized by neovascular AMD.^21,22^

### OCT Scanning and Measurement Protocols

**FIGURE 1:**
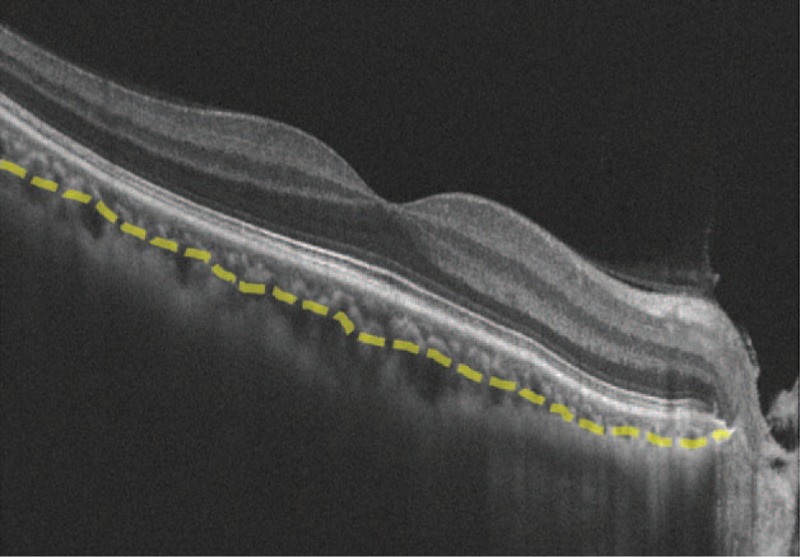
Representative choroidal B-scan image obtained from SD-OCT. Image shows manual segmentation of Sattler layer border and Haller layer (yellow line). SD-OCT = spectral-domain optical coherence tomography.

The choroidal images were obtained using the Zeiss Cirrus SD-OCT (Carl Zeiss Meditec, Inc.) with version 4000 software included. All measurements were performed by 2 independent masked trained examiners (LL, SX). Areas of conflict were discussed, and discrepancies were resolved by consultation with a third masked investigator. Subfoveal choroidal thickness (SFCT) was measured on the OCT screen, by using the contained Zeiss Cirrus SD-OCT Eye Explorer software (version 6.5). Thickness was measured vertically from the outer surface of the hyper-reflective line of the (retinal pigment epithelial) RPE/Bruch membrane band to the sclera-choroidal junction. The choroidal area (CA) was defined as the region between the outer RPE and inner scleral borders. Because of the limitations in identifying the choriocapillaris, the choroid was further subdivided into 2 layers. The Haller layer area (HA) was defined as the larger outer oval vascular profile, whereas the Sattler layer area (SA) was defined as a region between the outer RPE/Bruch membrane band and the inner line of the hypointense space, representing HA (Figure 1).^23–25^

The CA and SA were analyzed by 2 independent masked investigators (LL, SX), using the unit pixels Image Pro Plus 6.0 software.^25^ We performed enhanced-depth imaging (EDI)-OCT to improve the visualization of the hyper-reflective line between the HA and the sclera.^26^ Choroidal en face images were used to reconstruct en face views at 60 μm below the RPE/Bruch membrane, with a depth of 20 μm, where the inner choroid was well visualized in most healthy control eyes.^27^

Volume scans were calibrated to scan a square, 6 × 6 mm region of the posterior pole. The scan protocol for the macula region was a 512 × 128 raster scan, acquired in approximately 2.5 seconds.

### Statistical Analysis

Statistics were calculated using SPSS software (IBM SPSS, Version 20.0, IBM Corporation, Armonk, NY). *P* < 0.05 was considered statistically significant in this study. Continuous variables, such as age or SFCT, were described as mean ± SD. One-way analysis of variance (ANOVA) was used to compare age, choroidal thickness, and the percentage of area of the SA.

## RESULTS

### Subject Characteristics

The 154 eyes of 96 individuals, with a mean age of 67.1 ± 9.2 years (range 55–85 years), were included in this study, among which 32 eyes of 16 individuals were age-matched normal eyes included as controls. Twenty-six eyes of 20 individuals were fellow uninvolved eyes with intermediate/late AMD, 29 eyes of 20 individuals were early AMD, 28 of 18 individuals were intermediate AMD, and 39 of 22 individuals were late AMD. The age differences between the 5 groups showed no significant difference (*F* = 1.12, *P* = 0.35). Among 96 individuals included, 58.3% (56/96) were males and 41.7% (40/96) were females, 51.9% (80/154) were right-sided, and 48.1% (74/154) were left-sided. Fourteen fellow eyes underwent cataract surgeries, 15 eyes had a >3.0 diopters refractive error, and 9 eyes with histories of retinal detachment and vitrectomy were excluded (Table 1).

**TABLE 1:**
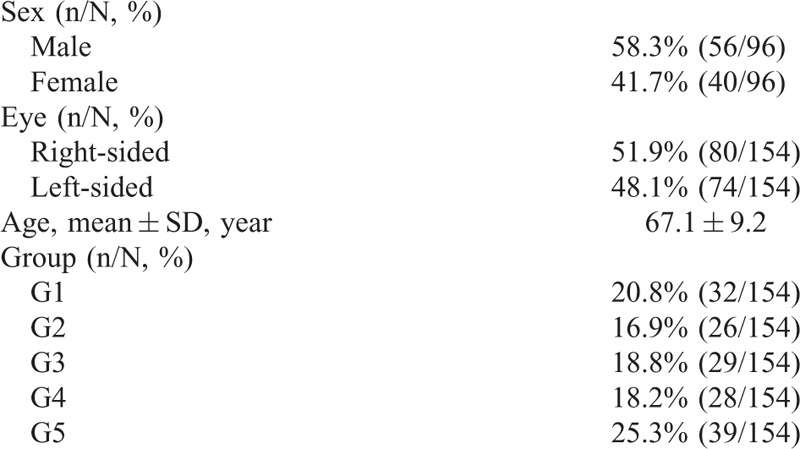
Clinical Characteristics of Included Patients

### Choroidal Findings

All subjects underwent measurement with SFCT, whereas 110 eyes underwent measurements of percentage of SA, among which 32 were in G1, 24 in G2, 28 in G3, and 26 in G4. Measurements of areas of SA in the eyes from the exudative AMD group were not able to be obtained, because the choroidal neovascular vessels (CNVs) showed hyper-reflection, and thus sheltered the signal from the SA and HA.

### Subfoveal Choroidal Thickness Changes

**FIGURE 2:**
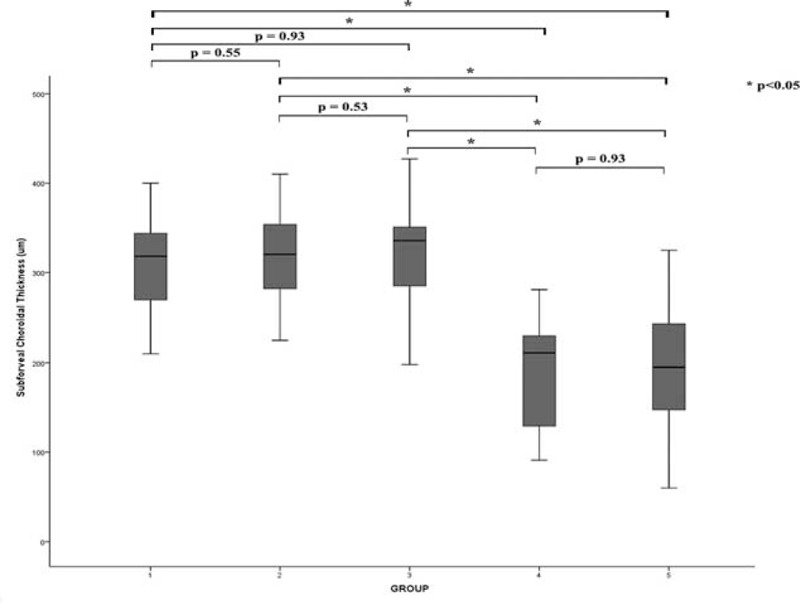
Box plots show differences in thickness of subfoveal choroid in 5 groups. Choroidal thickness in group 4 and group 5 were significantly lesser than in early AMD eyes (*P* < 0.001). AMD = age-related macular degeneration.

The mean SFCT was 295.4 ± 56.8 μm in age-matched normal eyes, 306.7 ± 68.4 μm in fellow uninvolved eyes of AMD patients, 293.8 ± 80.4 μm in early AMD patients, 215.6 ± 66.2 μm in G4, and 200.4 ± 66.6 μm in G5 (*F* = 14.2, *P* < 0.001). Though the thicknesses of SFCT measurements decreased from G1 to G3, no significance was found between these 3 groups (G1 vs G2, *P* = 0.55; G1 vs G3, *P* = 0.93; G2 vs G3, *P* = 0.53), or between intermediate AMD and late AMD groups (*P* = 0.93). However, choroidal thicknesses in the early AMD group (G3) were significantly greater than in the intermediate AMD group eyes (G4), and in the late AMD eyes (G5) (*P* < 0.001). Representative images are shown in Figure 2.

### Percentage of SA Area Changes

**FIGURE 3:**
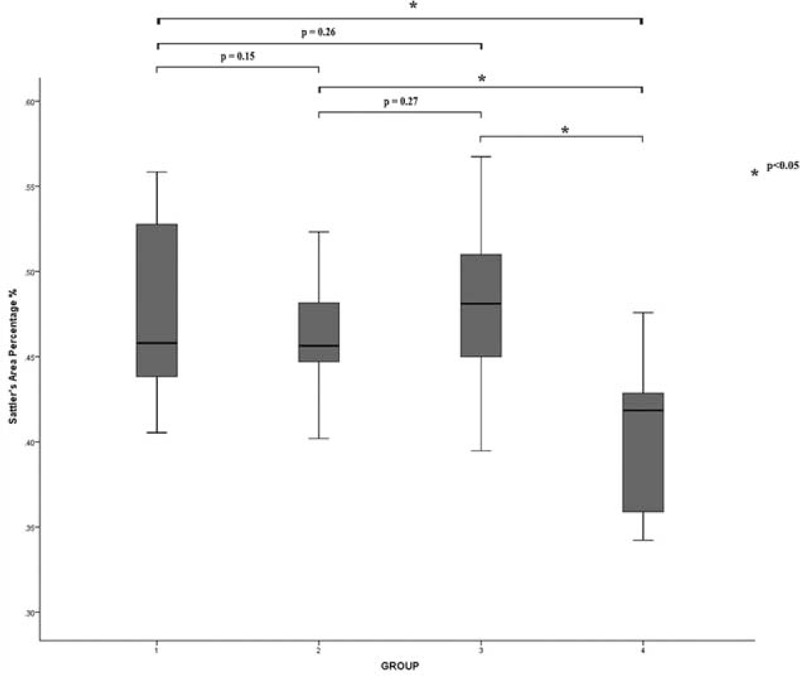
Box plots show differences in percentage of Sattler area (SA) in 4 groups. The mean percentage of SA in normal eye (G1) and fellow eye of immediate/late AMD (G2) and early AMD (G3) were significantly higher than the eye of intermediate AMD (G4). AMD = age-related macular degeneration.

**FIGURE 4:**
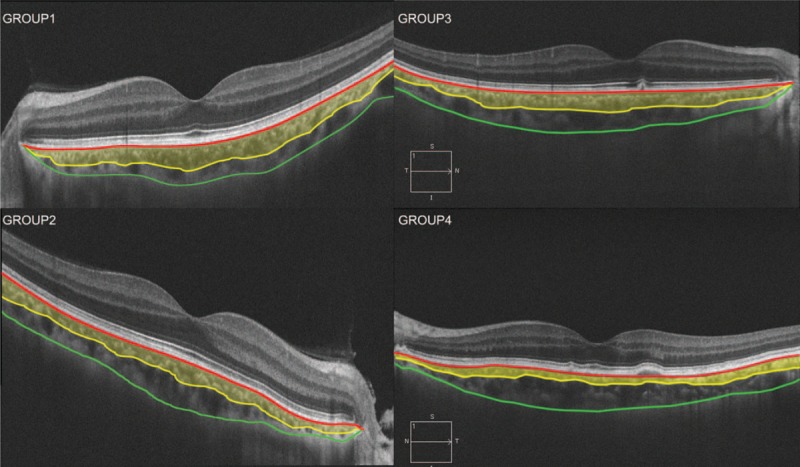
Example of choroidal B-scan images obtained from SD-OCT showing the percentage area of Sattler layer (yellow area) decreased from group 1 to group 4. SD-OCT = spectral-domain optical coherence tomography.

The mean percentage of SA of affected eyes in each group were 47.2% in G1, 45.8% in G2, 47.6% in G3, and 41.1% in G4 (*F* = 5.53, all *P* < 0.001). The mean percentage of SA in normal eyes (G1), fellow eyes of AMD patients (G2), and fellow eyes from early AMD patients (G3) were significantly higher than eyes of intermediate AMD patients (G4) (G3 vs G4, *P* < 0.001). There was no significant difference between G1, G2, and G3 fellow eyes (G1 vs G2; *P* = 0.15; G2 vs G3, *P* = 0.27; and G1 vs G3, *P* = 0.26, respectively) (Figures 3 and 4). The morphological features of choroidal findings in the 4 groups are summarized in Table 2.

**TABLE 2:**
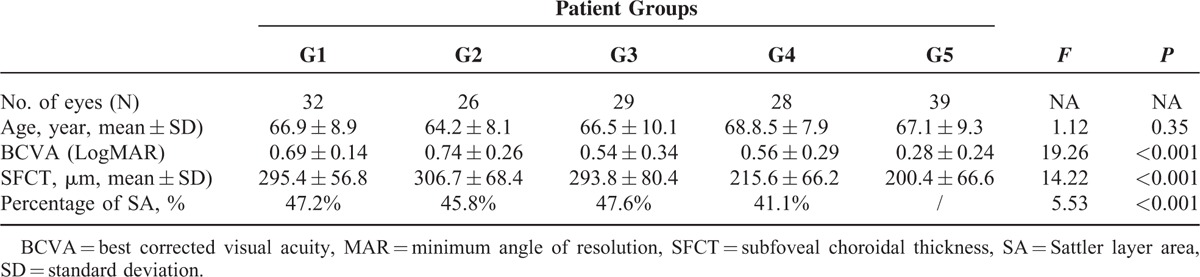
Morphological Features of Choroid in Patients

### En Face Enhanced Depth Imaging OCT changes

**FIGURE 5:**
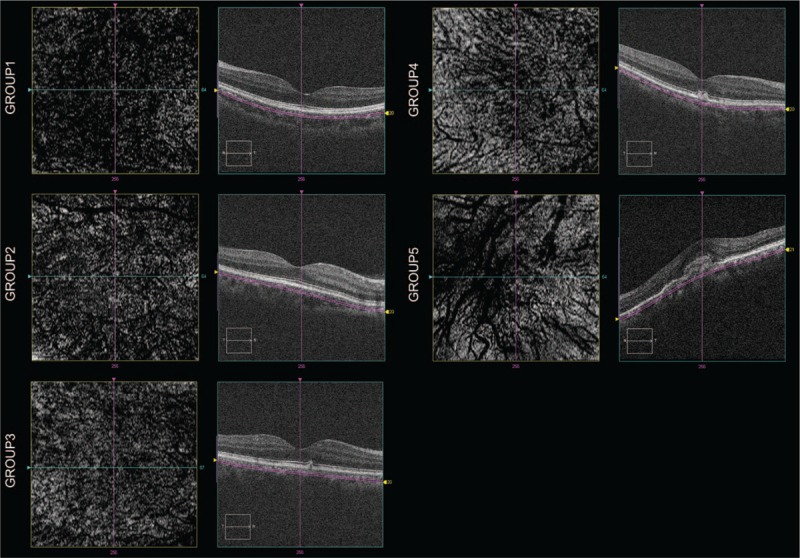
En face EDI-OCT scans of 6 × 6 mm subfoveal region of the 60 μm below RPE/Brunch membrane 20 μm in depth in 5 groups. Images show the dense distribution of dark regions in group 1 (top left), increasing bright-spotted hyper-reflective melanocytes in SA in group 2 and group 3, absence of small vessels in group 4, and almost complete absence of SA, straight large lumen vessels of Haller layer area in group 5. EDI-OCT = enhanced-depth imaging optical coherence tomography.

En face views were used to analyze the pattern, density, and size of choroidal vessels at 60 μm below the RPE/Bruch membrane band, with a 20 μm depth. Figure 5 illustrates the choroidal vascular patterns of the 5 groups. The en face image shows the dense distribution of dark regions, with small bright spots between them, in the age-matched normal eye group, representing small to medium-sized vessels and normal vascular network of the SA. Visibility of the vascular density in G2 and G3 was reduced and substituted by more bright spots, due to the hyper-reflection of melanocytes in the SA. The increased bright regions between the vessels, together with the absence of small vessels in the intermediate AMD group, showed thinning of the SA. While in the late AMD group, the SA was almost completely absent, and straight, large lumen vessels of HA were seen, indicating the atrophy of capillaries and medium-sized vessels of the choroid.

## DISCUSSION

The study measured the choroidal thickness and microstructural changes during the progression of AMD, including age-matched normal eyes, fellow uninvolved eyes of late/immediate AMD, and early, intermediate, and late AMD patient eyes, classified according to the consensus for clinical classification of AMD.^28^ The retina has a high metabolic demand for oxygen supplied by the underlying choroid.^29^ The blood flow of the choroid decreases during aging, mainly due to a decrease of blood volume, rather than velocity.^30^ With the development of SD-OCT for assessment of the choroid, the cross-sectional structure and segmentation of subvascular layers can be quantified with good reliability.^31^ Age-related changes in choroidal thicknesses have been documented in normal eyes and in various macular diseases.^32,33^ Some studies have confirmed that both the dry and exudative AMD patients had thinner than average choroids, compared with age-matched normal eyes.^34^ Some studies also reported reduced choroidal thickness in early AMD eyes,^35,36^ whereas others reported highly variable or no changes of choroidal thickness with early AMD.^16^ In our study, choroidal thickness was measured using SD-OCT and screening software for images at different stages of AMD, representing the progression of AMD. The SFCT measurements revealed decreases between age-matched normal eyes and early AMD eyes. Although no significant differences were found between these 3 groups, the mean SFCT was significantly thinner in intermediate AMD eyes and late AMD eyes, when compared with eyes before early-stage AMD. These results were in agreement with recent studies,^16,37^ indicating that the measurements of choroidal thickness, using OCT, might be good diagnostic criteria for AMD.

The SA is made up of medium-sized choroidal vessels, defined as thick, round, or oval-shaped hyper-reflective cores in the mid-choroid, also including the choriocapillaris, which is not easily distinguished from SA on OCT images.^23^ It was reported that the choroidal blood flow reduction was mediated by the underlying choriocapillaris supplied by SA as a “feeder vessel” layer,^38^ and choroidal thickness decreased during AMD progression, such as in the eyes with subretinal deposits or GA.^39^ The loss and/or decrease of the choriocapillaris and the medium-sized choroidal blood vessels were shown histologically in AMD patients.^17^ However, the relevance of SA changes to the pathogenesis of thinning of the choroid in AMD is poorly understood.

In our study, the percentage of SA changes were measured and significant decreases were found in the intermediate and late AMD patients, compared with AMD in early stages, whereas there was no significant difference between age-matched normal eyes, fellow uninvolved eyes of late/immediate AMD, and early AMD eyes. According to the evidence-based investigation in 2013, there was low risk of 5-year progression to late AMD for normal aging changes (0.5%) and early AMD (1.3%), whereas there was a 50% risk for the highest intermediate AMD risk group.^6^ The results in our study that there is differences between G1∼G3 and G4∼G5 is in line with the rate of progression to advanced AMD.

Though the percentage of SA and SFCT showed no significant changes, the microstructure of the choroid, using en face technology, showed reduced vascular density of the SA, and was substituted by bright spots of melanocytes from normal eyes to late AMD eyes. These results suggested that atrophy of the capillaries and medium-sized vessels may enable an earlier diagnosis, and could distinguish the changes in the perfusion of the sublayer blood flow of the choroid during the 5 stages of AMD.

The pathophysiological factors in AMD not only include changes in vascular components of the choroid, but also in lipofuscin accumulation in the RPE/Bruch membrane.^40^ There are 2 suggested mechanisms of AMD development, based on clinical and histologic studies. One is that the decrease in the ability of the choroid to deliver oxygen to the RPE and retina causes the damage, and the other is that the deposit of retinal tissues leads to choroidal neovascularization, owing to tissue accumulation in the RPE/Bruch membrane, and subsequent interference with the components that maintain the stability of the RPE-choriocapillaris, eventually resulting in choriocapillaris atrophy.^17,41^ It was showed that with extensive small drusen, pigment abnormalities, there is only a 1.3% probability of progression to advanced AMD by year 5, whereas the 5-year estimated probability of progression to advanced AMD with extensive intermediate drusen, large drusen was 18%.^42^ The results of this study demonstrated a relationship between the increasing size of drusen and decreasing thickness of the subfoveal choroid. The diffuse deposits of RPE/Bruch membrane, and the reduced density of the SA could eventually cause atrophy of the choroid.

Limitations of the study included the relatively small number of patients. Choroidal thicknesses and the dense vascular network of the inner choroidal in all SD-OCT images were evaluated manually, because of the lack of software for automated measurement, using the OCT equipment. Eyes presenting with GA were not included in this study because of the difficulty of patient recruitment. PCV eyes were also excluded because of the different pathophysiological features when compared with general wet AMD. Moreover, though no significant differences were shown in age and refractive errors between the 5 groups, eyes with refractive errors >3.0 diopters were excluded, because it was still a potential factor that could have influenced the choroid measurements.

In summary, our study revealed associated choroidal microstructural changes in AMD eyes. SFCT was reduced, mainly due to the reduced percentage of SA during progression of the disease. The microstructural changes in the capillaries and in medium-sized vessels of AMD eyes, when shown in en face images, occurred earlier than what was observed in SFCT. Our study provided a better understanding of AMD progression, using SD-OCT and the en face technology. Further studies are necessary to fully establish our findings as a diagnostic procedure.
